# Key Stakeholder Perspectives on Introducing a Front-of-Pack Labelling Scheme on Packaged Foods in China: A Qualitative Study

**DOI:** 10.3390/nu14030516

**Published:** 2022-01-25

**Authors:** Xuejun Yin, Lihong Ye, Xin Xin, Lin Xiang, Yue Yu, Ruijie Yan, Kehan Wen, Maoyi Tian, Alexandra Jones, Simone Pettigrew, Wei Liu, Yuexin Yang, Juan Zhang

**Affiliations:** 1 The George Institute for Global Health, University of New South Wales, Sydney, NSW 2052, Australia; xyin@georgeinstitute.org.au (X.Y.); ajones@georgeinstitute.org.au (A.J.); SPettigrew@georgeinstitute.org.au (S.P.); 2 School of Population Medicine and Public Health, Chinese Academy of Medical Sciences and Peking Union Medical College, Beijing 100730, China; yelihongpumc@163.com (L.Y.); xianglinpumc@163.com (L.X.); yanrj@sph.pumc.edu.cn (R.Y.); 3 Faculty of Psychology, Beijing Normal University, Beijing 100875, China; xin.xin@bnu.edu.cn (X.X.); 202028061054@mail.bnu.edu.cn (Y.Y.); 202028061047@mail.bnu.edu.cn (K.W.); wei.liu@bnu.edu.cn (W.L.); 4 School of Public Health, Harbin Medical University, Harbin 150088, China; 5 Chinese Nutrition Society, Beijing 100022, China; yxyang@263.net

**Keywords:** front-of-package nutritional labelling, food policy, China, stakeholder interview

## Abstract

Front-of-pack (FoP) labelling on foods is recommended by the World Health Organization (WHO) to address the growing global burden of diet-related noncommunicable diseases (NCDs), but this policy has not yet been implemented in China. The aim of this study was to ascertain key stakeholders’ views on barriers and facilitators to developing a feasible and acceptable FoP labelling policy in the Chinese context. Semistructured interviews were used to elicit opinions from diverse representatives in roles of FoP labelling policy influence. Participants were identified by purposive and snowball sampling. The Consolidated Framework for Implementation Research (CFIR) was adopted to facilitate data collection and analysis. Themes and subthemes were generated using deductive and inductive approaches. Thirty participants were interviewed. The major barriers were the absence of national contextual analysis, perceived complexity of the process of policy development, disagreement on a preferred FoP labelling format, cost for the food industry, low priority compared to food safety policies, lack of existing regulatory framework or authorised nutrient profiling system, limited knowledge of FoP labelling, and the lack of planning and engagement with stakeholders. Facilitators included existing prerequisites, experiences and lessons from the pilot, policy coherence with Healthy China 2030, and support from external agents (e.g., WHO). Further efforts are required to develop and collate evidence to demonstrate the scientific, legal, and political feasibility of introducing effective FoP labelling.

## 1. Introduction

Noncommunicable diseases (NCDs) are the leading cause of death both worldwide and in China [1,2]. Unhealthy diets are the major contributor to the surging global burden of obesity and diet-related NCDs [3,4,5]. Making food choices with adequate information holds the key to changing people’s eating patterns [6]. Food labels have traditionally consisted of information provided directly on packaging to assist decision making at the point of purchase, but may also include more recent efforts to convey information through technology such as “smart” or “virtual” labels [7]. Nutrition labelling is recommended as a policy tool through which governments can guide consumers to make informed food purchases and healthier eating choices [8]. To address the hazardous effects of dietary risk factors on NCDs, the Chinese government issued the General Rules for Nutrition Labelling of Packaged Foods (GB28050) in 2010, which were implemented as a mandatory standard in 2013. These rules require manufacturers to present quantitative information on the energy, protein, fat, carbohydrate, and sodium content of foods, and provide a list of ingredients on food packages [9]. Consumer understanding and use of this information on the back-of-pack remains very low [10]. To supplement this more detailed nutrition information, simple, graphical Front-of-Pack (FoP) labels have emerged as a feasible and acceptable strategy to facilitate healthier food purchasing [11]. The World Health Organization (WHO) recommends implementing FoP labelling as a “Best Buy” (i.e., cost-effective) intervention to combat NCDs [12].

Worldwide, at least 32 government-endorsed FoP labelling schemes have been implemented [12,13]. The currently operating FoP labelling schemes include “Multiple Traffic Light”, which indicates red (high), amber (medium), or green (low) levels of fats, sugars, and salt; “Health Star Rating”, which gives packaged food an overall score from 0.5 (least healthy) to 5.0 (most healthy) stars; “Nutri-Score”, which presents a coloured scale of A (green, higher quality) to E (dark orange, lower quality); octagonal “warning labels” that mark products as high in saturated fats, salt, sugar, or calories; and positive signposts such as the “Keyhole logo”, which can be displayed on products with less sugars and salt, more fibre and wholegrain, and healthier or less fat than food products of the same type. These schemes vary in legal status (voluntary/mandatory), format (reductive or interpretive), and their aim of either guiding consumers towards healthier options overall or directing them away from foods that exceed specified nutrient thresholds [14]. Reductive FoP labels provide factual information from the nutrition facts panel with little interpretation of this information [15]. In contrast, interpretive FoP labels include features that evaluate this information based on nutrient profiling. Nutrient profiling serves as the scientific basis to classify or rank foods according to their nutritional composition for reasons related to preventing disease and promoting health [16]. From existing literature, FoP labels that are interpretive are more effective at directing consumers towards healthier choices than reductive FoP labels, because interpretive FoP labels provide more convenient, readily understood nutrition information, and lead to more accurate impressions of product healthfulness than reductive FoP labels [17]. The China National Food Industry Association and China Nutrition Society issued an industry-initiated interpretive FoP labelling scheme in 2018, which promoted the use of a “Healthier Choice” or “Smart Choice” logo on the front of packages of foods that contain a low level of total fat, sodium, and sugar according to an association-developed standard (Supplementary Figure S1) [18]. “Healthier choice” logos were only applied to specific food categories, including cereal, bean, dairy, nut, meat, egg, vegetable, and fruit products, while “Smart Choice” was used for beverages and other snack products, such as puffed products, jelly, and creamy products. Both labels were voluntary tools with limited adoption [19]. 

Healthy China Movement prioritises the promotion of healthy diets with goals of cutting dietary salt, oil and sugar nationwide by 2030. It encourages the food industry to provide supplemental nutrition information on the front of packages to help consumers identify healthier food options [9]. At the global level, World Health Organization (WHO) published guiding principles and a framework manual for FoP labelling to assist Member States in developing and implementing FoP labelling in 2019 [20]. Research on the feasibility of developing FoP labelling in China is limited. Qualitative methods are helpful in examining feasibility and determining the perceptions of stakeholders. We conducted a qualitative study to identify factors that may influence the process of FoP labelling development in the Chinese context. Findings from this study were assessed against the WHO manual to outline the next key steps in FoP labelling development in China. 

## 2. Materials and Methods

A qualitative research design was deployed to seek stakeholders’ perspectives on developing and implementing the FoP labelling policy in China. The Consolidated Criteria for Reporting Qualitative Research guidelines, which covers the reporting of studies using interviews and focus groups, guided reporting of the study results [21]. 

### 2.1. Study Participants and Sampling

The aim of quantitative sampling approaches is to draw a representative sample from the population, so that the results of studying the sample can then be generalised back to the population. However, the aim of doing qualitative research is to capture rich and in-depth information. Improved understanding of the phenomenon of interest is more important than the generalisability of results. Included stakeholders were those who could provide in-depth information about the readiness for FoP labelling implementation in China. We drew upon recognition in the WHO’s Guiding Principles and Framework Manual for FoP Labelling and the literature on best-practice FoP labelling that a wide range of stakeholders have an interest in FoP labelling development and implementation. In the initial conceptual stages, informal engagement from a wide group of experts is recommended to gain an understanding of the likely issues and possible opposition to FoP labelling [13,20]. In this study, we included government agencies from both health sectors (e.g., National Health Commission) and regulatory sectors (e.g., State Administration for Market Regulation). Technical support agencies were the institutes affiliated with the National Health Commission and are actively involved in generating evidence in food and nutrition policy development and implementation (e.g., National Institute for Nutrition and Health, Chinese Centre for Disease Control and Prevention). Professional associations were those nongovernment institutes related to food, nutrition, and consumers (e.g., China Nutrition Association). Media representatives included experts in communicating science, influential nutrition bloggers in social media, and people working in a nonprofit scientific organisation engaged in communicating science-based information on food safety, and nutrition and health (e.g., China Food Information Center). Industries included multinational food companies, domestic food companies of varying size, and supermarket retailers. We selected three groups of consumers who often buy packaged foods to capture their attitudes to FoP labelling. Three groups of consumers were young adults who purchased packaged food in their daily life (ages 21–25), mothers with children aged 3–12, and office workers who like snacks (ages 31–35). The triangulation of stakeholder groups can improve the credibility and transferability of our study results. 

To identify groups of individuals that are especially knowledgeable about FoP labelling and nutrition policy in China and to reach target participants who are not easily accessible, purposive and snowball sampling methods were applied. Potential participants were identified through the following three steps. First, an initial list of potential participants was formed from the 2020 China Nutrition 30 Forum list of attendees [22], members of the Advisory Board of State Nutrition Plan (2017–2030), and experts who participated in the amendment of nutrition labelling standards [23]. Second, individuals were grouped by their organisation. Potential participants were then purposively selected using factors such as job position, experience, and professional expertise. Third, during the interviews, participants were asked to name others who were proficient in this field and likely to contribute to the generation of in-depth knowledge. To retain the stakeholders’ anonymity, their roles but not organisations are presented in in this paper.

In every case, participants were contacted directly by telephone or email by a senior member (JZ) of the research team to seek their participation. If participation was declined, we then identified an alternative participant with a similar position in the same category of organisation. The participants’ contact information was identified either through existing networks or publicly available institutional/organisational websites. Participants were provided with a participant information sheet and informed about the purpose of the study and their right to withdraw from the study at any time. They were asked to sign an informed consent form prior to the commencement of their interview.

### 2.2. Data Collection

Data were collected through face-to-face individual in-depth interviews. The interview guides were developed based on the Consolidated Framework for Implementation Research (CFIR). CFIR is a meta-theoretical framework that synthesises constructs from a range of theories about dissemination, innovation, organisational change, implementation, knowledge translation, and research uptake. It comprises five domains: (1) the characteristics of the intervention (i.e., the advantages of implementing FoP labelling, the evidence for implementing FoP labelling, and cost); (2) the outer setting (i.e., perceived population need for FoP labelling and policy influences); (3) the inner setting (i.e., priority of FoP labelling in implementing organisations, compatibility with current workflow, and resources available for implementation); (4) the characteristics of individuals (i.e., knowledge and self-efficacy of how to use FoP labelling); and (5) the process of implementation (i.e., planning and engaging). The interview guides were tailored to different stakeholder groups to reflect their areas of expertise (Supplementary Table S1). Our research team consisted of researchers in nutrition, food policy, qualitative methodology, psychology, and PhD students. All interviews were carried out in person by researchers with experience in qualitative research and in-depth interviews (J.Z., X.X., both are professors, female) with at least two note-takers (L.Y., R.Y., L.X., all are Ph.D. students) at the interviewee’s office or a private space to allow interviewees to share their views freely and confidentially without being influenced by others. A visual methodology was also used in the interview to enhance research credibility [24]. It involved participants being provided with photos of packaged foods that had various FoP labels superimposed on the front of the packs. Examples of FoP labels used elsewhere internationally (e.g., healthy choice logos, warning labels, traffic lights, Health Star Ratings) were presented. Photo elicitation facilitated discussion and enabled the interviewer to obtain a clear understanding of the interviewees’ attitudes and beliefs relating to FoP labelling. All interviews were conducted in Mandarin Chinese. The sample size was determined by information saturation, which occurred when no new information about FoP labels emerged during the interviews.

### 2.3. Data Analysis

Data analysis occurred concurrently with data collection. Data analysis of early interviews was conducted before or during the recruitment and interviewing of later participants. The audio-recorded interviews were transcribed verbatim and then coded using both inductive and deductive methods. First, transcripts were independently read by two team members (X.Y., L.Y.) to identify preliminary codes (inductive approach). Second, codes with similar meanings were clustered to form subthemes and themes, and then linked to relevant theoretical constructs in the CFIR (deductive approach). The coding book was finalised by constant comparison until no new concepts emerged and all conceptual codes were linked to CFIR domains. Throughout, coders met to review coding, conduct team debriefing meetings, and reach consensus on code names and meanings. Data were analysed in Chinese. Only emerging themes and associated illustrative quotations were translated into English. This process involved forward-translation (from the source language into English) and back-translation (from English to their source language). Both versions were compared to check accuracy and equivalence. Any discrepancies were discussed between the two bilingual translators (X.Y., L.Y.). Typical quotations were used to support the interpretations presented in the results. To maintain stakeholders’ confidentiality, we provided the stakeholder group and study identification number at the end of each cited quotation without any identifiable information. Coding and analysis were conducted in NVivo (version 12). We decided to use the Nvivo as analysis software because it works well with thematic analysis and allows coding comparison between different coders [25].

## 3. Results

All invited stakeholders agreed to be interviewed. In total, 30 stakeholders were interviewed before reaching data saturation. These individuals represented government agencies (*n* = 5), technical support agencies (*n* = 8), professional associations (*n* = 3), the food industry (*n* = 7), the mass media (*n* = 3), and consumer groups (*n* = 4). The average time of the interviews was 66 ± 27 min. Factors influencing the development and implementation of FoP labelling policy spanned 16 constructs across five domains of the CFIR. Subthemes emerging under the CFIR major domains are summarised in Table 1.

### 3.1. Intervention Characteristics

The main constructs that emerged under this domain included: (1) evidence strength and quality, (2) relative advantages/disadvantages, (3) adaptability, (4) trialability, (5) complexity, (6) design quality and packaging, and (7) cost. 

The relevant advantage compared to the current nutrient declarations, lessons learnt from the pilot of “Healthier Choice” or “Smart Choice” FoP labelling, and trialability were identified as facilitators that would influence the success of FoP labelling development. Most participants agreed that an interpretive FoP label format would be easier for consumers to understand than nutrient declarations. Regarding the relative advantages/disadvantages of FoP labelling compared with other interventions to promote healthier diets, government representatives thought education campaigns would be more effective in achieving consumer behaviour change than FoP labels.

*“I think, from the consumer’s point of view, the simpler the better. For example, the percentage and values in the nutrition facts panel are too complicated. I won’t read it nor understand it. If there is a logo, I think it is recognized by national experts, and I can rely on it”.*

(Technical support agency, 0208)

Lessons learnt from the “Healthier Choice” or “Smart Choice” program were seen as potentially informing the adoption of a new FoP labelling design. Participants suggested that a pilot program within several food categories or in selected cities prior to the formal launch nationwide could be used to demonstrate feasibility. Participants from the food industry and government agencies discussed the need for a transition phase between policy release and implementation.

Insufficient evidence was identified as one of the potential barriers to FoP labelling. Examples of noted evidence gaps included: the contribution of excess sodium, sugar, and fat in packaged foods to the burden of diet-related disease in China; the effectiveness of FoP labels in driving behaviour change; the need for a validated nutrient profiling system to underpin FoP labels; and cost-effectiveness modelling studies of the risks and benefits associated with FoP labelling implementation in China. 

Participants suggested that existing FoP labelling schemes in other countries need to be tailored to the Chinese context. There was disagreement among stakeholders about the optimal format for a FoP labelling scheme. Supporters of reductive FoP labels thought implementation would be more feasible than for interpretive FoP labels. They assumed that reductive FoP labels could be considered more acceptable by the food industry, a finding that was echoed by food industry representatives. They also expected that the process of implementing reductive FoP labels would be less complex as those objective numbers do not require other calculations than the existing nutrient reference values. Others disagreed on the basis that it would reproduce information already available on the back of the pack and still be difficult and time-consuming for consumers to interpret. In terms of the interpretive schemes, government representatives expressed a preference for warning labels that can provide consumers with an explicit warning on unhealthy package foods. By contrast, representatives from the food industry vigorously opposed warning labels because they would negatively impact their brands and sales. The food industry representatives were also concerned about extra costs, including investment in reformulation, nutritional analysis of products, and changing product packages to incorporate FoP labelling. These costs were noted to be potentially more challenging for small- and middle-sized food companies.

*“If FoP labelling is implemented, we will face increased costs. We need to input in nutrition analysis, and food packages need to be re-made”.*

(Food industry, 0403)

### 3.2. Outer Setting

Public needs and resources, peer pressure, and external policies and incentives were identified as important outer setting constructs. Increasing government attention on the growing burden of NCD, alignment with the goal of Healthy China 2030, and external support from WHO were regarded as outer setting facilitators. Health-sector stakeholders proposed FoP labelling as an essential policy for responding to the epidemic of obesity and other diet-related NCDs in China. Many participants mentioned that Healthy China 2030, one of the most important initiatives of the Chinese government, provided a unique opportunity to introduce FoP labelling policies to achieve the goal of reducing the population-level consumption of salt, sugar, and fat. In addition, existing FoP labels in many countries might accelerate the progress of China’s commitment to FoP labelling.

*“The initiative of Healthy China 2030 is a good opportunity for the development of FoP labelling in China. It would be better if the aim of FoP labelling can be clarified in the 14th Five-Year Plan”.*

(Government 0105)

Several policy-level barriers were identified. No participants believed that FoP labelling would be adopted by the food industry if a voluntary approach was adopted in China. A mandatory approach was highly recommended by government representatives, but it was not clear how FoP labelling would be incorporated into current legal frameworks and nutrition policies. For example, there is currently no authorised nutrient profiling system to define healthy or unhealthy products. In addition, representatives from government agencies were concerned that FoP labelling regulations and monitoring strategies might conflict with existing policies supporting small business development.

*“As a regulatory authority, we’d better not put our fingers in another’s pie. What we can do is those mandatory by laws and regulations. The FoP labelling currently is not mandatory. It is beyond the responsibility of our department”.*

(Government 0103)

*“The current policy orientation of the country is to encourage the development of enterprises. It is difficult to introduce any policy that increases the burden on enterprises”.*

(Government 0105)

### 3.3. Inner Setting

Emerging subthemes in the inner setting domain reflected constructs of networks and communication, compatibility, relative priority, available resources, and access to knowledge. Subthemes identified as facilitators included existing nutrition analysis techniques, the high compatibility with health sectors’ broader aims, and support from sections of the food industry that intend to produce low-sodium, low-sugar, low-fat foods. 

China has sufficient laboratory capacity for various methods of analysing sodium, sugar, and fat in packaged foods. Government representatives from the health sector strongly supported the development of FoP labelling as it was perceived as being consistent with their organisations’ goal of supporting the implementation of the Healthy China Strategy. FoP labelling was also described as consistent with the business strategy of some international food industry stakeholders. These industry representatives already accepted FoP labelling elsewhere, and some reported that large companies had started to invest in reformulation.

*“In fact, some large companies may support it. Such as Nestlé. They have their own nutrition science department to do related research and product development. As for as I know, they have their own nutrition profiling”.*

(Technical support agency, 0207)

Low priority and a lack of multi-sectoral cooperation mechanisms were identified as barriers within the inner setting. Government representatives noted that the priority of food policy was still on food safety rather than over-nutrition. Health education was identified as the preferred strategy by all interviewees. They argued that individuals have freedom of choice and bear responsibility for their health, and they expressed the belief that health education can empower individuals to make better choices.

*“The focus is still to improve the nutritional literacy of consumers through health education. Otherwise, even if the government and industry agree to make great efforts to introduce FoP labelling, consumers still do not know how this information can help them make choices. Such a policy is far ahead of the consumers’ perception and will not help them much”.*

(Professional association, 0301)

### 3.4. Individual Characteristics

The main relevant construct in this domain related to knowledge and beliefs about FoP labelling. Low knowledge of FoP labelling appeared to be a barrier. Government representatives who had limited knowledge about the effectiveness of FoP labelling interventions were not convinced of the need to develop an appropriate FoP labelling policy in China. The interviewed consumers had little knowledge about FoP labelling. They would like to refer to the FoP labelling while making purchase choices.

*“I do not know the meaning of this (FoP labelling) logo. When I buy food, I only pay attention to when is the production date and its ingredient list. This logo is not published, and I do not know its standard”.*

(Consumer, 0504)

### 3.5. Process 

The decision-making process was described as complex and involving multiple sectors at different levels. Participants noted that feedback from stakeholders should be solicited and incorporated during all stages of the planning process. Objections from some opinion leaders working on food policies were seen to be the major barrier in the process of developing FoP labelling policy. The main reason for the objection was the concern that introducing FoP labelling would potentially unduly increase regulation on the food industry and impede profits and growth. Other opposing opinions included the perceived risks of losing flexibility and posing negative reputational impact on the whole food industry.

*“They feel that the market is over-regulated now. Adding FoP labelling to the existing standards would worsen the over-regulation, which will suppress the industry’s enthusiasm and restrict the industry’s vigorous development. Nevertheless, we believe that chronic diseases are now dramatically increasing in China, especially adolescent obesity. We are not talking about cracking down on the industry, but we want to urge the industry to produce healthier food”.*

(Government 0104)

Convincing opinion leaders to introduce FoP labelling, engaging academic researchers to provide strong country-specific evidence, formally appointing implementation leaders in government agencies and technical support agencies, activating advocates, and getting support from external agencies were suggested as facilitators under the construct of engaging stakeholders. Some participants mentioned that the WHO has been actively advocating for FoP labelling in China and thought this could potentially influence FoP labelling development in a desirable direction.

*“The government can issue a document to support the development of FoP labelling. But the important thing is to appoint someone to implement it step by step. Currently, the technical support agencies’ attitude towards developing nutrient profiling system for different food categories is not clear”.*

(Government, 0104)

## 4. Discussion

This study identified significant barriers and facilitators to the introduction of FoP labelling in China. These are discussed below in the context of previous research and the WHO’s Guiding Principles and Framework Manual for FoP Labelling [20] to identify the major steps that need to be taken to develop and implement a FoP labelling scheme in China.

### 4.1. Contextual Analysis and Knowledge Synthesis to Inform FoP Labelling Policy Design

According to the WHO Guiding Principles, contextual analysis is the first step in developing a FoP labelling scheme [20]. Our study findings indicated a belief by key stakeholders that there was a lack of formative evidence to provide the rationale for proceeding with the development of FoP labelling in China. Stakeholders were not convinced by the current evidence that a large proportion of the NCD burden in China is attributed to packaged food. Epidemiological analyses of diet-related NCDs and the dietary patterns of the Chinese population should be undertaken to inform FoP labelling development and guide implementation. The increased production of processed food, rapid urbanisation, and changing lifestyles have led to a shift in dietary patterns in China. Chinese packaged foods have been found to have higher saturated fat, total sugars, and energy density compared to those in Western countries [26,27]. FoP labelling can inform people about products that contain excess sodium, sugar, and harmful fats, and are therefore contributing to morbidity and mortality in the Chinese population. There were various levels of understanding of FoP labelling across stakeholders, with many exhibiting low awareness of existing global evidence for the effectiveness, acceptability, and implementation of these labels. Many key stakeholders would benefit from access to knowledge synthesis of the global evidence on FoP labelling in tandem with the development of national evidence to support the development of a national FoP label. Communication and dissemination of relevant research are needed to obtain stakeholders’ commitment to policy change. 

Both government and food industry representatives were concerned about potential industry costs associated with the introduction of FoP labelling. The human and economic losses associated with diet-related NCDs should be seriously considered as a counterpoint to these concerns. An economic evaluation of FoP labelling in Germany suggested that FoP labelling has the potential to substantially avert disability-adjusted life years, reduce treatment cost, prevent productivity losses, and reduce economic burden [28]. Arguments around the adverse economic impact, limiting freedom of choice, and personal responsibility raised by the food industry stakeholders were similar to the findings from Brazil and France where these have been made as part of attempts to delay or forestall the development of FoP labelling [29,30]. More work is needed to effectively address the opposition to FoP labelling, including by establishing cost-effectiveness modelling to estimate the risks and benefits associated with FoP labelling in the Chinese context.

### 4.2. Legal Framework for FoP Labelling

A major consideration for participants was the regulatory framework for FoP labelling scheme adoption, including whether the scheme should be mandatory or voluntary. The pilot of voluntary “Healthier Choice” and “Smart Choice” labelling in China demonstrated poor performance. Without legal incentives for uptake, the food industry is unlikely to adopt a voluntary FoP labelling scheme that has the potential to negatively affect consumer purchases. Evidence from countries that have implemented voluntary schemes shows that the food industry selectively avoids applying FoP labelling to products where they do not receive favourable ratings [31,32]. The stakeholders were unclear about the legal frameworks available in China to facilitate development of FoP labelling. There is an opportunity to follow standard procedures for legislative development by updating the existing China national standard for food safety and nutrition labelling policy (GB28050-2011) to support Healthy China 2030 [9]. Early engagement with legal experts, including those working for government, can support the development of a robust legal framework for implementing FoP labelling, particularly in the event of food industry opposition [13].

### 4.3. FoP Labelling Format and Nutrient Profiling System 

The results of this study indicate divergent opinions on the selection of FoP labelling formats among stakeholders. The WHO recommends that the format of FoP labelling (i.e., design and content) should be determined based on the aims and scope of the policy as determined by government [20]. The formats currently in use around the world can be classified as those aiming to discourage consumption of foods high in critical nutrients (i.e., warning labels) and those seeking to encourage healthier choices (i.e., Health Stars, Nutri-score, Traffic lights) by signalling both healthier and less healthy foods overall. In China, the aim and scope of FoP labelling have not been clearly set by government. Large-scale, cross-sectional surveys across culturally diverse countries have shown that FoP labelling format is related to effectiveness, and that consumer outcomes can differ by country [33,34]. Different FoP labelling designs in use worldwide can be tested to see which are most likely to achieve China’s aims. Given the differing preferences on label formats expressed by participants of this study, it is important that policy development be based on scientific evidence and protected from commercial conflicts of interest. 

Nutrient profiling is used to underpin FoP labelling policies [16,35]. The results of this study indicate that the perceived lack of a credible, authoritative nutrient profiling system is likely to be a fundamental barrier to implementing FoP labelling in China. Many nutrient profiling systems have already been developed worldwide, with some having been used for application in food labelling [36]. The selection of an appropriate nutrient profiling system depends on the labelling format chosen. Some require just one threshold per nutrient (i.e., warning labels), while others require multiple thresholds (e.g., traffic lights). A nutrient profile system aiming to score the nutritional quality of packaged foods in China has been developed by a nongovernment agency [37]. Validation studies and government endorsement of the nutrient profiling system may help its potential application. 

### 4.4. Consumer Health Education Campaigns

One major barrier identified in our study was the low demand for FoP labelling among consumer participants due to low knowledge of nutrition and FoP labelling. This echoes the results of previous surveys conducted among Chinese consumers showing a low awareness of the amount of sodium in packaged food and limited knowledge of how to use sodium labels [10,38]. An international systematic review of nutrition label education studies found that consumer health education could positively impact consumers’ label understanding and use [39]. FoP labelling itself is an education tool by providing nutritional information on food products. Improving the availability of FoP labelling and the capacity to effectively use FoP labelling can allow individuals to become more health-literate and nutrition-literate [40]. Ultimately, food companies can be incentivised to manufacturing healthier products as a result of changing consumer preferences and product choices [41]. 

### 4.5. Strengths and Limitations

There are several strengths to this study. First, it provides valuable insights into the situational country context, thereby contributing original knowledge to inform the development of a FoP labelling scheme in China. Second, the participants were from a range of different sectors involved in this issue, enabling the capture of multiple and divergent perspectives. Third, a qualitative methodology allowed the generation of in-depth, rich data, providing detailed descriptions and enabling understanding of perspectives. The design and analysis were guided by the CFIR framework, which has been previously used to review facilitators and barriers for nutrition interventions and policy development [42]. The main limitation of this study is that the data may lack generalisability due to limited sample size. However, attention has been paid to describing the research setting, methods, and interviewees to allow readers to consider likely transferability. Participants were recruited from national levels. Opinions expressed herein do not represent people from subnational levels who will be involved in implementation. Lastly, our study is an exploratory study using a qualitative approach. Quantitative studies are necessary to investigate the knowledge of and attitudes towards FoP labelling across the broader population to assess the potential impact of FoP labelling on food consumption and public health in the future.

Future research needs to focus on promoting the establishment of a legal framework under which FoP labelling would be introduced in China. The evidence gaps identified in this study reflected stakeholders’ uncertainties about the mechanisms and effectiveness of FoP labelling implementation and concerns about the potential impact on the economy. To build on our findings, more research is needed to provide evidence to support the development and implementation of FoP labelling to support policymakers in navigating these processes.

## 5. Conclusions

FoP labelling has received growing attention from Chinese government officials and technical support agencies and can be a potential strategy to tackle the challenge of diet-related NCDs. However, introducing a FoP labelling scheme in China was not perceived by the key stakeholders involved in this research to be feasible with the current evidence and legal framework. This study identified the next steps needed to develop a FoP labelling scheme in China. Policy development, which is led by the government and based on formative research that engages stakeholders appropriately while managing conflicts of interest, is most likely to lead to an acceptable, credible, and effective FoP labelling system to ensure maximal utility with the target population.

## Figures and Tables

**Table 1:** Themes under CFIR constructs by facilitators and barriers.

CFIR Constructs		Facilitators	Barriers
Intervention characteristics	Evidence strength and quality	“Healthier Choice” or “Smart Choice” as pilot	Lack of national evidentiary basis for developing FoP labelling policy
	Relative advantages/disadvantages	Perceived relative advantage compared with nutrition declaration	Perceived relative disadvantage compared with health education
	Adaptability		The need to adapt FoP labelling format
	Trialability	Trialability with phrase-in implementation	
	Complexity		Perceived complexity of developing, implementing, and monitoring FoP labelling system
	Design quality and packaging		Disagreement in FoP labelling format
	Cost		Extra cost for food industry
Outer setting	Patient needs and resources	Large burden of NCDs in China	Lack of customer demand
	Peer pressure	Peer pressure from other countries	Lack of legal framework
	External policies and incentives	Encouragement from Healthy China Action	Potential conflicts with existing policies
		Food safety law (GB28050) as fundamental policy	
Inner setting	Networks and communications		Lack of multi-sectional communication
	Compatibility	Consistent with the health sector’s goal	
		Consistent with the development goals of some international and large companies	
	Relative Priority		Food safety is priority in food policy
			Health education is the main nutrition-related intervention
	Available Resources	Having mandatory nutrient declaration on food back packages as a prerequisite for FoP labelling	Lack of authoritative nutrient profiling system for Chinese food categories
Individual characteristics	Knowledge and Beliefs about the Intervention		Low knowledge about FoP labelling
			Concern about bringing misunderstanding to consumers
			Concern about negative impact on company’s reputation and sales
Process	Planning and engaging	Convincing opinion leaders to introduce FoP labelling	
		Appointing implementation leader/organisation	
		Activating domestic advocators	
		Engaging academic researchers to provide strong evidence	
	External change agents	Advocacy and support from external change agents (i.e., WHO)	

## Data Availability

All the data and materials of this qualitative study are available from the corresponding author on reasonable request.

## Supplementary Materials

The following supporting information can be downloaded at: https://www.mdpi.com/article/10.3390/nu14030516/s1, Table S1: Healthy Food Policy Analysis in China-Society/ Association/Government agency. Figure S1, “Healthier Choice” or “Smart Choice” logo on the front of packages of foods.
