# Supplementary material for: Key Stakeholder Perspectives on Introducing a Front-of-Pack Labelling Scheme on Packaged Foods in China: A Qualitative Study
Source: Nutrients. 2022 Jan 25;14(3):516. doi: 10.3390/nu14030516 (PMC8840240; doi:10.3390/nu14030516)
Supplement: Supplementary file 1 [file nutrients-14-00516-s001.zip › nutrients-1454238-supplementary/suppl-Table S1.pdf]

Table S1. Healthy Food Policy Analysis in China-Society/ Association/Government agency

Introduction 5mins	Dear expert/madam/sir:<br>Hello! We are the research team of the School of Population Medicine and Public Health, Chinese Academy of Medical Sciences & Peking Union Medical College. We are currently doing research on "Healthy Food Policy Analysis in China". In this interview, we mainly want to know your views on views on the need for a FoP labelling scheme and understand barriers and facilitators to developing a feasible and acceptable FoP labelling policy. All the content of this interview is only used for this research, and we guarantee that the information is only used for internal use and will never be leaked. Please take a look at the informed consent form for this interview. Do you agree that we will record this interview? Thank you very much for your support and help to this research in your busy schedule!		
	**CFIR domains**	**Question**	**Make a detailed inquiry**
15mins	Intervention characteristics And Individual characteristics	There are several kinds of food nutrition labels. Could you please tell us about their usefulness and the current situation in the market?	/
		Displaying food identification Toolkits display	
		Have you participated in the formulation of a certain nutrition-related policy, can you share it? (E.g. process, difficulties, unforgettable things encountered)	
		What do you think is the difference between FOP and the nutrition label you know before?	
	Inner setting	What work in your department is related to FOP? How is this work done? What difficulties have you encountered in the past? How was it solved?	What difficulties did your department/institution encounter in the process of participation?
		If we want to make the FOP standard into a national NPS, what do you think we need to do?	/

15mins	Process and inner setting	What do you think are the working steps (paths) to promote the FOP policy? What tasks (functions) do these departments undertake?	How do these departments cooperate, communicate, and dialogue? How influential are these departments?
		In the process of promoting FOPL, what are the roles/responsibilities of your organization?	Follow-up: Please tell us about the current progress, difficulties encountered, and potential solutions?
		What technical conditions are needed in the early stage of Fopl's introduction? Who will provide it?	What help does the NPS of the Nutrition Society provide? What else needs to be improved?
		The introduction of FOP will inevitably require financial support. Which aspects do you think need financial support? How can we get financial support?	Who will provide the funds?
		In order to promote the introduction of FOPL, how should companies conduct publicity, science popularization, and advocacy? How to promote and popularize science for consumers? (Take food label as an example)	Who will be responsible for the promotion?
5mins	Outer settings	What existing policies conflict with FOP?	/
		Regarding FOP practices abroad, what do you think are worth learning from domestically?	
5mins	Process	In your opinion, after the introduction of FOP, which supervisory departments and what work can they do to ensure the smooth implementation of the policy?	The introduction of policies may bring losses to some companies. What measures do you think we can take to win the support of food companies?

			Which agency or department is required to be responsible/cooperate to solve? How do you cooperate with regulatory authorities and what difficulties have you encountered? How to solve it?
		The introduction of policies may bring losses to some companies. What measures do you think we can take to win the support of food companies?	/
5mins	Characteristics of individuals	What do you think FOP can help the public to develop healthy eating habits?	/
5mins	Inner setting and outer setting	In your opinion, what are the Strengths and Weaknesses for introducing nutrition policies including FOP?	From the perspective of policy environment, technical conditions, food companies, cross-departmental cooperation, policy implementation supervision, funding, etc.
		In your opinion, what are the Opportunities and Threats to the introduction of nutrition policies including FOP?	From the perspective of policy environment, technology, enterprise, cross-departmental cooperation, supervision and implementation, funding, etc.
Concluding remarks	Thank you very much for your support for this interview. The information you gave us today will promote the research and release of FOP in China. The information from this interview will also be applied to this research. If some information needs to be supplemented later, can we? Call back again? Thank you again!		
