# Supplementary figures and images for: Key Stakeholder Perspectives on Introducing a Front-of-Pack Labelling Scheme on Packaged Foods in China: A Qualitative Study
Source: Nutrients. 2022 Jan 25;14(3):516. doi: 10.3390/nu14030516 (PMC8840240; doi:10.3390/nu14030516)

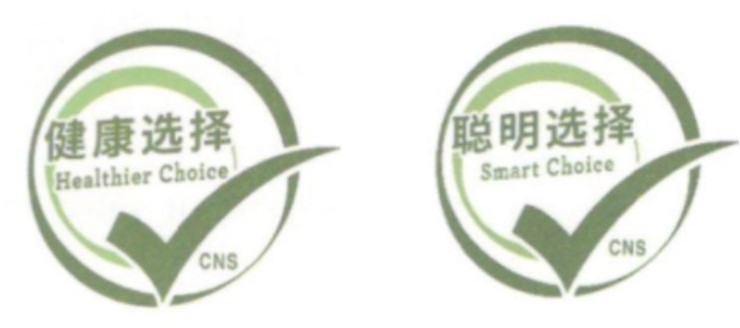

Supplement: Supplementary file 1 [file nutrients-14-00516-s001.zip › nutrients-1454238-supplementary/supplementary-Figure S1.jpg]
